# Effect of central sensitization on dizziness-related symptoms of persistent postural-perceptual dizziness

**DOI:** 10.1186/s13030-022-00235-4

**Published:** 2022-03-07

**Authors:** Kazuaki Hashimoto, Takeaki Takeuchi, Takayuki Ueno, Shunsuke Suka, Miki Hiiragi, Maya Yamada, Akiko Koyama, Yuzo Nakamura, Jun Miyakoda, Masahiro Hashizume

**Affiliations:** Department of Psychosomatic Medicine, Toho University School of Medicine, Tokyo, Japan

**Keywords:** Functional dizziness, Psychosomatic disorder, Central sensitization, Medically unexplained symptoms

## Abstract

**Background:**

Persistent postural-perceptual dizziness (PPPD) is a chronic functional dizziness symptom triggered by psychological stress, but its pathophysiology is unknown. Central sensitization is considered the cause of functional diseases, such as medically unexplained symptoms, and is a psychosocially affected condition. However, the association between dizziness symptoms in PPPD and central sensitization remains unclear. Thus, we conducted a cross-sectional study on the relation between dizziness symptoms and central sensitization in PPPD.

**Methods:**

We recruited 61 outpatients with dizziness who met the PPPD diagnostic criteria. In addition to the evaluation of dizziness symptoms using the Dizziness Handicap Inventory, the participants were evaluated using the Hospital Anxiety and Depression Scale, Pittsburgh Sleep Quality Index, and Central Sensitization Inventory (CSI). A CSI score of 40 or higher was defined as central sensitization syndrome (CSS), and the severity of each condition in CSS and non-CSS participants was compared. We also evaluated the association between dizziness symptoms and central sensitization and coexisting symptoms using linear multiple regression analysis.

**Results:**

We analyzed the data of 50 valid responses (valid response rate of 82.0 percent). Compared with the non-CSS group, the CSS group had a higher degree of disability owing to dizziness and a higher rate of complications of anxiety and depression. The regression analysis results showed that the severity of central sensitization was a related factor that could enhance the dizziness symptoms of PPPD.

**Conclusions:**

Central sensitization may affect the dizziness symptoms of PPPD as an exacerbating factor.

## Background

Dizziness is one of the most common clinical symptoms. In primary care, the frequency of dizziness symptoms is as high as 5 percent [1], although its cause is often unknown, despite proper testing. A systematic review showed that up to 80 percent of patients cannot identify the cause of dizziness [2]. Dizziness is also associated with psychiatric factors, particularly with depression [3] and anxiety [4]. Poor sleep quality is also associated with the exacerbation of dizziness symptoms in chronic dizziness cases involving psychological factors [5]. In recent years, dizziness characterized by a shift from the preceding dizziness symptoms to a chronic condition owing to psychological stress has been treated as a disease concept, called persistent postural-perceptual dizziness (PPPD) [6]. PPPD has psychosomatic aspects, but the mechanism of chronic dizziness symptoms has not been elucidated.

Physical symptoms that cannot be explained medically are called medically unexplained symptoms (MUS); they are reported to have a prevalence of about 25 percent to 75 percent [7]. MUS is a physical symptom that can appear with or without organic disease [8], and complaints of physical symptoms range from headaches to dizziness. Psychological factors are often involved in the physical symptoms of MUS, and the effectiveness of psychotherapy [9] and antidepressants [10] has been reported. Some symptoms in MUS may be associated with central sensitization, a neurophysiological condition that induces hyperalgesia owing to the hyperexcitability of the central nervous system [11]. Central sensitization is a pain-related concept, but it cannot be measured directly, so quantitative sensory tests are used for inference [12]. However, they are difficult to operationalize in clinical practice because they are too costly, and central sensitization is assessed by CSI [13]. The CSI scores have been reported to correlate with pain-modulatory system function assessed by the conditioned pain modulation task [14], and with quantitative sensory tests [15]. Central sensitization that greatly affects the physical condition is called central sensitization syndrome (CSS), which is reported in many areas of disease, including gastrointestinal and neurological disorders. Specifically, CSS has been reported to be associated with 10 diseases, such as tension-type headache, migraine, irritable bowel syndrome, rheumatoid arthritis, and fibromyalgia [11].

Central sensitization in MUS has recently been implicated in symptoms other than pain. For example, a large cohort study in which central sensitization was assessed using CSI reported that it predicted fatigue with or without pain [16]. In addition, several diseases associated with central sensitization have reported an association between central sensitization and dizziness. In fibromyalgia, desensitization of pain transduction pathways results in excessive sensory input from lower limb muscles, and fibromyalgia patients are prone to loss of balance at rest [17]. However, patients with fibromyalgia perceive excessive wobbling rather than actual body sway, and it may be that abnormalities in depth perception signals through the spinal dorsal horn and cognitive processing of vestibular sensations may be impaired by central sensitization [18]. It has been reported that dizziness may also be induced in vestibular migraine by the mechanism of sensitization of self-motion perception by dysfunction of the vestibular nuclei [19].

Although the mechanism of PPPD is unknown, overadaptation of equilibrium function caused by a combination of factors such as visual and somatosensory dysfunction, anxiety, and fear has been proposed as a hypothesis [6]. Therefore, we hypothesized that the mechanism of PPPD involves the same association between dizziness and central sensitization in MUS, where the processing function of the vestibular senses is impaired by central sensitization, thereby exacerbating dizziness. However, no studies have examined the relation between dizziness symptoms in PPPD and central sensitization. Thus, in this study, we conducted a cross-sectional study on the relation between dizziness symptoms and central sensitization in PPPD.

## Methods

### Participants

**Fig. 1:**
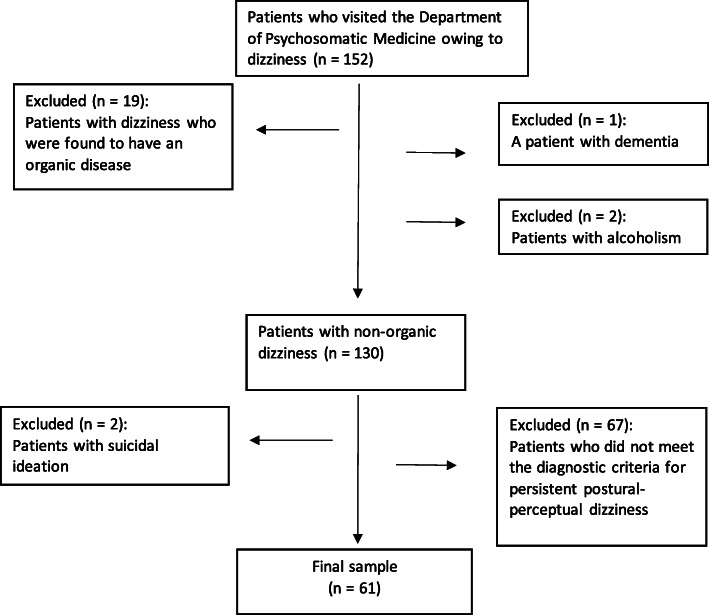
Flowchart of participant recruitment

Between January 2019 and November 2020, 152 patients aged 20 to 79 years visited the Department of Psychosomatic Medicine, Toho University Medical Center Omori Hospital, for dizziness. A total of 61 cases that met the diagnostic criteria for PPPD [6] were included in the study. We referred to previous reports [10, 20] regarding the diagnosis of PPPD. The patients were pre-examined by a general practitioner and an otolaryngologist with general laboratory tests, such as imaging tests, electronystagmography, posture tests, and balance tests. We excluded 19 patients with vertigo who had obvious organic factors, one patient with dementia, and one patient with alcoholism from the study. We also excluded two patients with suicidal ideation and 67 patients who did not meet the diagnostic criteria for PPPD, leaving us with a final sample of 61 patients (Fig. 1).

### Questionnaires

The Dizziness Handicap Inventory (DHI) [21] was used to evaluate dysfunction owing to dizziness. The DHI is a questionnaire consisting of 25 questions on the impairment of living function caused by dizziness. The scores range from 0 to 100 points, with a score of 46 or higher being evaluated as severe [22]. The reliability and validity of the Japanese version of the DHI have already been verified [23].

The central sensitization inventory (CSI) [24] was used to evaluate central sensitization. CSI consists of Part A (CSI-A), which assesses the severity of subjective symptoms common to CSS, and Part B, which determines the history of CSS-related disorders. For CSI-A, over 40 points is the cutoff value used for discriminating CSS [25]. The Japanese version has been confirmed to be reliable and valid [13].

We evaluated anxiety and depression using the seven-item Hospital Anxiety and Depression Scale (HADS) [26]. The anxiety and depression scales are scored from 0 to 21 points, and each scale has an optimal cutoff value of 8 points or more in general practice [27]. The Japanese version has been confirmed to be reliable and valid [28].

We used the Pittsburgh Sleep Quality Index (PSQI) [29] to assess insomnia. The PSQI is an 18-item questionnaire that evaluates sleep disorders experienced in the past month. The scores range from 0 to 21 points, with 6 points indicating a high possibility of sleep disorder. The Japanese version has been verified for reliability and validity [30].

### Outcome and data analysis

Cases with a CSI-A score of 40 points or more were treated as having CSS [25]. We compared the background factors of the CSS and non-CSS groups and the number of participants above the cutoff of each questionnaire. The following were considered in the comparisons using the chi-squared test: sex; medical therapy: antidepressant use, benzodiazepine use; insomnia: PSQI score of 6 points or higher; anxiety: HADS anxiety scale score of 8 points or higher; depression: HADS depressive scale score of 8 points or higher. Comparisons of the presence or absence of drinking and smoking habits and marriage status were evaluated using the Fisher’s test. Student’s t-test was used to compare age and years of education, and the Mann-Whitney U test was used to compare treatment and disease duration.

To clarify the relation between dizziness symptoms and central sensitization in PPPD, we performed linear multiple regression analysis with DHI as the dependent variable. The independent variables were the CSI-A, PSQI, and HADS scores, whereas the background factors that showed differences between the CSS and non-CSS groups were used as adjusting factors.

All statistical analyses were performed with the EZR Ver 1.32 statistical package [31]. Two-tailed *P*-values less than 0.05 were considered statistically significant.

## Results

Of the 61 participants with PPPD, 11 had some missing data: fifty provided valid responses, giving a valid response rate for this study was 82.0 percent. Fifteen cases (30.0 percent) were defined as having CSS.

Table 1 shows the diseases reported by the participants prior to developing PPPD. All of the participants had developed some predecessor disease prior to the onset. The most common diseases were generalized anxiety disorder, benign paroxysmal positional vertigo, and panic disorder. Multiple diseases preceded in some of the participants.

**Table 1:** Diseases that preceded the onset of PPPD in the subjects (n = 50)

Diseases	
Otolaryngological disorders	15(30.0%)
Benign paroxysmal positional vertigo	8(16.0%)
Meniere’s disease	4(8.0%)
Cholesteatoma	1(2.0%)
Vestibular neuritis	1(2.0%)
Sudden deafness	1(2.0%)
Mental disorders	37(74.0%)
Generalized anxiety disorder	9(18.0%)
Panic disorders	8(16.0%)
Somatoform disorder	7(14.0%)
Depressive disorders	6(12.0%)
Adjustment disorder	6(12.0%)
Hypochondriasis	1(2.0%)
Neurological disorder	9(18.0%)
Migraine	5(10.0%)
Tension headache	2(4.0%)
Meningitis	2(4.0%)
Others	3(6.0%)
Orthostatic dysregulation	2(4.0%)
Premenstrual Syndrome	1(2.0%)

Table 2 presents a comparison between the CSS and non-CSS groups. A comparison of background factors showed that the CSS group included more smokers. No significant differences were found for the other factors. Regarding the comparison of questionnaire scores, which were the evaluation items, the mean DHI score of the CSS group was 55.6 points, while the mean DHI score of the non-CSS group was 32.2 points. The CSS group included more participants whose HADS and DHI scores exceeded the cutoff values, compared with the non-CSS group. Meanwhile, the CSS and non-CSS groups showed no clear difference regarding the PSQI cutoff value being exceeded.

**Table 2:** Patient Characteristics (n=50)

	Non-CSS(n=35)	CSS(n=15)	*P* value
Sex			0.99
Male	12(34.3%)	5(33.3%)	
Female	23(65.7%)	10(66.7%)	
Age(years ±SD)	56.2 ±15.1	48.6 ±14.8	0.11
Habit			
Smoking	3(8.6%)	6(40.0%)	0.02
Drinking	8(22.9%)	4(26.7%)	0.99
Education(year)	13.1 ±2.6	14.2 ±2.7	0.16
Married	26(74.3%)	8(53.3%)	0.19
Disease duration(month)	11.0 [5.0-24.0]	11.0 [6.0-22.0]	0.96
Treatment duration(month)	9.0 [4.0-30.0]	9.0 [5.5-24.5]	0.92
Medical therapy			
Antidepressant	17(48.6%)	6(40.0%)	0.95
Benzodiazepines	15(42.9%)	9(60.0%)	0.42
Questionnaire(cut-off)			
DHI($\geqq $46)	9(25.7%)	9(60.0%)	0.04
HADS Anxiety($\geqq $8)	5(14.3%)	12(80.0%)	<0.001
HADS Depression($\geqq $8)	9(25.7%)	11(73.3%)	0.004
PSQI($\geqq $6)	20(57.1%)	12(80.0%)	0.22

*DHI* dizziness handicap inventory, *HADS* hospital anxiety and depression scale, *PSQI* Pittsburgh sleep quality index

Values are given as mean (±standard deviation) or median (inter quartile range)

Table 3 shows the results of linear multiple regression analysis with DHI as the dependent variable and CSI-A, PSQI, HADS anxiety and depression scales, and smoking as the independent variables. Smoking, which was the difference in patient background between the CSS and non-CSS groups, was selected as an adjustment factor. CSI-A was extracted as a factor that enhanced the severity of dizziness in PPPD. The other dependent variables were not significantly associated with DHI. There was no apparent multicollinearity among the dependent variables.

**Table 3:** Multiple linear regression analysis of Dizziness Handicap Inventory (n=50)

Independent variable	*β*	Standard error	t value	*p* value	VIF
CSI-A	0.59	0.24	2.39	0.02	2.44
HADS-anxiety	-0.17	1.02	-0.17	0.86	4.16
HADS-depression	1.62	0.84	1.93	0.06	3.41
PSQI	0.27	0.61	0.44	0.65	1.15
Smoking	-4.02	7.29	-0.55	0.58	1.22

*CSI* central sensitization inventory, *HADS* hospital anxiety and depression scale, *PSQI* Pittsburgh sleep quality index, *β*: standardized regression coefficient, *VIF* variance inflation factor

Multiple R^2^ = 0.48, adjusted R^2^ = 0.42

## Discussion

We compared the clinical features of the CSS and non-CSS cases of PPPD who visited the psychosomatic medicine department at our hospital. Dizziness symptoms were stronger in the CSS group than in the non-CSS group. The CSS group also had a higher distribution of smokers and greater anxiety and depressive symptoms. Results of the multiple regression analysis revealed the effect of central sensitization as a factor that exacerbated the dizziness symptoms of PPPD.

A difference in DHI scores of 18 points or more is generally reported as a clinically meaningful change in dizziness patients [22]. The mean difference in DHI scores between the CSS and non-CSS groups was 23 points, which is greater than the 18 points considered to be a clinically significant change. In other words, not only were there statistical differences, but from a clinical point of view, the CSS group may have more severe dizziness symptoms than the non-CSS group. The severity of dizziness symptoms in the CSS group among PPPD participants is similar to the results of previous studies in MUS. According to research on MUS, the group with the symptom pattern of central sensitization is the group with the most severe clinical symptoms [32]. Similar to MUS, the dizziness in PPPD suggests that organic abnormalities may not fully explain the disease state, but that central sensitization may enhance clinical symptoms.

Smoking, in addition to being associated with anxiety and depression [33], has also been associated with pain, and chronic smoking in particular has been reported to be associated with central sensitization [34]. In some patients with CSS, smoking has been reported to be associated with the onset and exacerbation of pain, depression and psychological states [35, 36]. As a physiological factor, nicotine is known to have effects on the central nervous system, including dopaminergic neurotransmitter systems [37] and endogenous opioid system [38]. In addition, a bidirectional relationship has been reported in which pain further motivates smoking [39]. Furthermore, anxiety and depression are already known to be associated with CSS [25, 40]. The fact that the CSS group in our study was a sample with more smokers and more anxiety and depression than the non-CSS group is consistent with these previous studies. Central sensitization has been suggested to be a mechanism mediating anxiety and depression [41], which is consistent with our results where only central sensitization remained as an associated factor in the linear multiple regression analysis.

In central sensitization, the mechanism for suppressing non-essential sensations is disrupted by a dysregulation of descending nociceptive reception [11, 42]. Several studies have reported somatosensory amplification and impaired perception and processing of external stimuli in MUS [43, 44]. Furthermore, as mentioned in the introduction, central sensitization has been associated with dizziness in MUS [17–19]. One of the causes of chronic dizziness is the sensory gating system [45], a mechanism that controls peripheral sensory information to the cerebral cortex, might cause dysfunction owing to chronic stress [46]. From this study alone, it is not clear what level of vestibular sensory processing is impaired by central sensitization in PPPD, but it may be that chronic stress sensitizes the vestibular senses and inappropriately processes sensory information, thereby enhancing the dizziness symptoms of PPPD.

### Strengths and limitations

This study is the first to examine the relation between dizziness symptoms, central sensitization, and multiple psychological factors in the unexplained mechanism of PPPD. A previous study has reported that about 30 percent of PPPD cases have mental illness as a precursor to PPPD [6]. However, about 70 percent of our participants had psychiatric disorders as a precursor to PPPD.

This study was conducted by a single medical institution specializing in psychosomatic medicine at a university hospital, which may have biased the characteristics of our sample. Or in other words, PPPD may have subgroups regarding the onset mechanism, and the results of our study might have indicated the characteristics of the subgroups. It is also possible that covariates that may affect dizziness and central sensitization have not been observed. Patients with dizziness symptoms whose organic abnormalities are not clear are generally difficult to treat; this is one of the reasons the mechanism has not been elucidated. One of the strengths of this study is that we were able to consider central sensitization and multiple psychological factors at a specialized institution for psychosomatic medicine. Our results suggest that central sensitization may influence vertigo symptoms in some PPPD patients, thus psychosomatic medicine may be an important aspect in the treatment of PPPD. In addition, based on CSS and DHI scores, the detection power in this study was 80 percent or more, indicating a certain validity, although the sample size was small. Future research should consider collaborations among many research facilities and using a larger sample size.

## Conclusion

This study investigated the factors related to the dizziness symptoms of PPPD. We found a positive association between DHI and CSI-A scores in PPPD, even after accounting for anxiety, depression, and smoking. The results also suggested that the coexistence of CSS in PPPD may increase the degree of disability attributable to dizziness symptoms in patients.

## Data Availability

We are not able to share the current study data because sharing data is not permitted by our hospital ethics committee.
